# Progress in Pseudotyping Lentiviral Vectors Towards Cell-Specific Gene Delivery In Vivo

**DOI:** 10.3390/v17060802

**Published:** 2025-05-31

**Authors:** Ariana Arduini, Harshita Katiyar, Chen Liang

**Affiliations:** 1 Lady Davis Institute, Jewish General Hospital, Montreal, QC H3T 1E2, Canada; ariana.arduini@mail.mcgill.ca (A.A.); harshita.katiyar@mail.mcgill.ca (H.K.); 2 Department of Medicine, McGill University, Montreal, QC H3G 2M1, Canada; 3 Department of Microbiology and Immunology, McGill University, Montreal, QC H3A 2B4, Canada

**Keywords:** lentiviral vectors (LVs), tropism engineering, targeted gene delivery, in vivo gene therapy, pseudotyping, host restriction factors

## Abstract

Lentiviral vectors (LVs) have become a fundamental tool in gene therapy due to their unique ability to transduce both dividing and non-dividing cells, transfer large genes of up to 10 kb, and facilitate stable, long-term expression of therapeutic genes into target cells. A key application of LVs is the ex vivo genetic modification of patient-derived cells, such as the production of CAR-T cells by transducing isolated T cells with LVs to express the CAR gene, enabling them to target and destroy cancer cells once infused back into the patient. However, these ex vivo gene therapy drugs are often dismally unaffordable due to the complex procedures involved, including cell isolation, genetic modification, and expansion, along with the significant risks associated with immune conditioning to ensure successful engraftment. To overcome these barriers, direct in vivo transgene delivery to physiologically relevant cells has been explored, bypassing the need for ex vivo manipulations and reducing costs. Yet, a major challenge in this approach is engineering LV cell tropism to ensure the precise targeting of specific cells while avoiding off-target effects. Recent advances in modifying LV surface proteins have shown promise, including the successful in vivo generation of CAR T cells and ensuing clinical trials. This review is aimed at providing an up-to-date account of the progress in engineering LV tropism, covering the utility of different heterologous viral envelopes and their engineering to achieve cell-type-specific delivery and host immune evasion, and highlighting the potential of in vivo gene therapy to improve the affordability and accessibility of life-saving treatments.

## 1. Introduction

The recent years have seen inspiring successes in gene therapy, offering hope for the treatment of diseases deemed otherwise incurable by traditional medicine. A pivotal moment came in 2016 with the approval of the first gene therapy drug, Strimvelis (Orchard Therapeutics), by European Medicines Agency (EMA) for the treatment of adenosine deaminase (ADA)-deficient severe combined immunodeficiency (SCID), a life-threatening monogenic disorder affecting infants. Strimvelis involves the infusion of autologous hematopoietic stem and progenitor cells (HSPCs) that are genetically modified with a gammaretroviral vector to express a functional ADA gene [1,2]. In 2017, the United States Food and Drug Administration (FDA) approved its first gene therapy drug, Kymriah (Tisagenlecleucel, developed by Novartis), for the treatment of relapsed or refractory B-cell acute lymphoblastic leukemia (ALL) in children and young adults [3,4]. Kymriah utilizes an ex vivo approach to genetically modify autologous T lymphocytes with a lentiviral vector (LV) that expresses an anti-CD19 chimeric antigen receptor (CAR), which are subsequently reinfused into ALL patients to target and eliminate leukemia cells. As of the end of 2024, the FDA had approved an additional 43 cellular and gene therapy products for a wide range of diseases, including myeloma, bladder cancer, ALL, hemophilia, B-cell lymphoma, sickle cell disease, Duchenne muscular dystrophy, aromatic L amino acid decarboxylase deficiency (AADC), retinal dystrophy, among others [5].

Despite these remarkable successes of gene therapy, significant challenges remain. One major hurdle is the high cost of gene therapy drugs, which often limits access for those who need them the most. For example, the recent discontinuation of Pfizer’s hemophilia B gene therapy drug, Beqvez, which is priced at USD 3.5 M, underscores the economic barriers to the widespread adoption of gene therapy treatments [6,7]. One solution is the targeted in vivo delivery of therapeutic gene products to physiologically relevant cells. This can be partially accomplished through localized injection into relevant tissues or organs, such as the eye or the brain. For example, the gene therapy product, Luxturna, is given via subretinal injection to treat retinal dystrophies with RPE65 mutations [8]. Another route is respiratory delivery, as demonstrated in pre-clinical studies where inhaled gene therapy products achieved durable expression of the cystic fibrosis transmembrane receptor in 9–15% of lung epithelial cells [9,10]. In contrast, intravenous delivery poses greater challenges due to rapid dilution and hepatic clearance, although this liver tropism has been leveraged to treat hepatic diseases like hemophilia [11]. Ultimately, developing targeted vectors that can deliver therapeutic genes precisely to physiologically relevant cells in vivo offers a fundamental strategy to improve efficacy and reduce the overall cost of gene therapy [12]. In the context of CAR T cell therapy, such vectors would eliminate the need for complex ex vivo cell manipulation prior to reinfusion into patients, as well as the requirement for bone marrow myeloablation and conditioning [13,14]. Additionally, targeted delivery vectors would greatly minimize off-target effects and reduce potential side effects, potentially cutting the costs and enhancing safety. For example, the cost of in vivo CAR T cell therapy could be as low as USD 5000 per dose if successful, and several companies have already begun or planned clinical trials for in vivo CAR T candidates, some of which utilize LVs [15].

Significant progress has been made in engineering LVs with specific cell tropism by redesigning virus surface glycoproteins using modular, programmable approaches. This review aims to provide an up-to-date account of these advancements in designing cell-specific ligands to guide LVs to their target cells. Additionally, the review highlights strategies to enhance the LV transduction efficiency in primary cells by overcoming innate immune barriers, paving the way for more precise, effective, and accessible in vivo gene therapies.

## 2. The Utility of Lentiviral Vectors in Gene Delivery

LVs are one of the most commonly used viral vectors for gene delivery. Among viral vectors, retroviral vectors (including LVs) and adeno-associated virus (AAV) vectors are most frequently employed in the development of gene therapy drugs [16,17,18]. Retroviral vectors offer the advantage of integrating transgenes into the host genome to achieve permanent transgene expression. However, this integration capability also poses risks, including insertional mutagenesis which can lead to oncogenesis. For example, early trials with gammaretroviral vectors to treat X-linked severe combined immunodeficiency (SCID-Xl) resulted in leukemia in 4 out of 10 patients due to insertional oncogenesis [19,20]. Fortunately, LVs have demonstrated a relatively safer integration profile, reducing this risk [21,22]. In contrast, AAVs do not integrate into the host genome but can persist as episomal DNA, enabling long-term transgene expression [23]. Still, the use of AAVs also presents challenges, including pre-existing antibodies in the human population, which can severely reduce the efficacy of AAV-based gene therapies. In addition, AAVs have a relatively limited DNA packaging capacity (<5 kb) compared to LVs which can carry transgenes of up to 10 kb. Importantly, LVs are able to transduce non-dividing, quiescent cells which represent the main primary target cells in vivo for gene therapy [24]. Meanwhile, nanoparticles have rapidly emerged as a powerful non-viral alternative for gene delivery [25]. Nanoparticles offer advantages such as ease of modification, scalable production, and low antigenicity. However, they cannot fully replace viral vectors in many applications, partly due to their inability to achieve permanent gene expression, a key characteristic of retroviral vectors.

**Figure 1:**
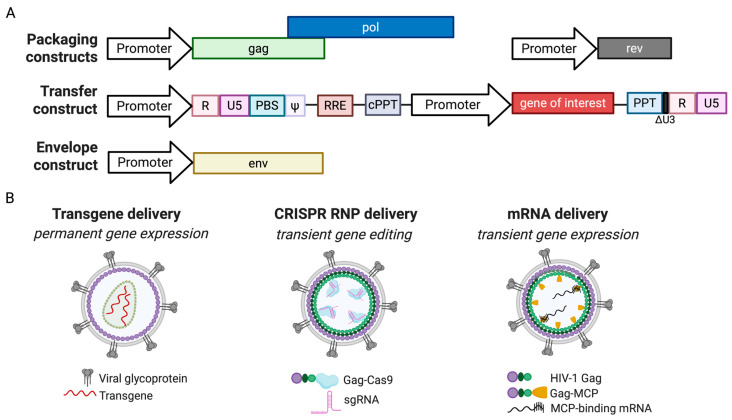
Lentiviral vector system and gene delivery strategies. (**A**) Schematic representation of components for LV production, including packaging, transfer, and envelope constructs. The packaging construct expresses viral Gag and Gag-Pol proteins to form virus particles, the transfer construct produces transgene RNA to be packaged into viruses, and the envelope construct expresses virus surface proteins to enable virus entry into target cells. ψ, RNA packaging signal. PBS, primer binding site. PPT, polypurine tract. cPPT, central PPT. (**B**) LV-based delivery approaches. LVs can be used for transgene delivery (permanent gene expression), mRNA delivery (transient gene expression), and CRISPR RNP delivery (transient gene editing). MCP, MS2 capsid protein. Created in BioRender.com; accessed on 9 May 2025.

LVs are derived from human immunodeficiency virus type 1 (HIV-1), the human retrovirus responsible for acquired immunodeficiency syndrome (AIDS). Infectious HIV-1 particles are formed by viral Gag and Gag-Pol proteins, which incorporate the viral envelope (Env) glycoprotein onto the virus surface and package two copies of the viral genomic RNA [26,27]. The LV system thus consists of three key components that together reconstitute virus particles capable of delivering transgenes into target cells (Figure 1A) [28]. The first component is the packaging construct, which expresses viral Gag and Gag-Pol proteins. Gag drives virus particle assembly, while Pol contains the viral protease, reverse transcriptase, and integrase. The viral protease cleaves Gag and Gag-Pol to release mature viral proteins, the reverse transcriptase converts the transgene RNA into DNA, and integrase mediates the integration of this DNA into the host genome within the nucleus [26,27,29]. Significant modifications have been made to the packaging constructs to minimize the number of viral genes and reduce the risk of generating replication-competent viruses. The second component is the transfer vector construct carrying the therapeutic genes. This construct also includes the viral RNA packaging signal (ψ), essential for the selective packaging of transgene RNA into LVs via interaction with viral Gag, as well as the sequences necessary for reverse transcription, such as the primer binding site (PBS), poly-purine tract (PPT), and repeat (R) region [30]. The PBS is where viral reverse transcription initiates from a cellular tRNALys.3 by annealing to the 18-nucleotide-long PBS. The R region mediates newly synthesized negative-strand DNA transfer from the 5′ to 3′ end of the viral genome. The PPT initiates the synthesis of positive-strand viral DNA [30]. There is also a copy of the PPT near the center of the viral genome called the central PPT (cPPT), serving as an additional start site of plus-strand DNA synthesis [31]. The Rev response element (RRE) is also inserted into the transfer vector to enhance the expression of transgene RNA with co-expression of the Rev protein. Upon binding to the RRE, Rev accelerates RNA export through engaging the Crm1 pathway [32]. The long-terminal repeat (LTR) has the U3, R, and U5 regions, and drives the expression of vector RNA. To enhance LV biosafety, self-inactivating (SIN) transfer vectors have been developed which lack the U3 region of the LTR, thus preventing the activation of nearby cellular genes after integration [33]. Furthermore, HIV-1 genes such as *tat*, *vif*, *vpr*, *vpu*, and *nef* have been removed from the LV system to retain only the minimal components required for viral packaging and transduction [34]. The third component is the envelope construct, which expresses viral surface glycoproteins that determine LV tropism and mediate entry into target cells. While this component is a key focus of this review, a detailed description of LV biology can be found in relevant reviews [35,36].

LVs have been primarily used to deliver transgenes for stable expression in target cells. For instance, they are widely employed for the ex vivo manufacturing of CAR T cells, where LVs deliver and stably express the CAR or T-cell receptor (TCR) genes [37]. Additionally, the FDA-approved gene therapy drug, Lyfgenia, utilizes LVs to express the functional HbA^T87Q^ gene in hematopoietic stem cells from sickle cell disease patients [38]. Beyond enabling stable gene expression, LVs have also been adapted for transient CRISPR-Cas9 delivery in the form of virus-like particles (VLPs) that do not carry transfer vector RNA [39] (Figure 1B). The VLP-based strategy thus does not involve reverse transcription and integration of the gene to be delivered, and has recently been employed for the safe delivery of CRISPR gene editors [40]. For example, engineering Cas9 to fuse with the C-terminus of Gag allows transient delivery of Cas9/gRNA, significantly lowering the risk of off-target editing [41,42,43,44]. In this approach, since the transfer vector is unnecessary, Gag can be significantly truncated to retain only its VLP-forming function, leading to the development of simplified enveloped delivery vesicles (EDVs) [45]. HIV-based VLPs have also been engineered to deliver mRNA for protein production by attaching RNA-binding proteins, such as the MS2 coat protein (MCP), to Gag or viral Vpr. This allows the selective packaging of mRNA containing the MS2-binding site into LVs, and enables transient protein production in target cells [46]. For instance, MCP-Vpr has been used to successfully deliver SaCas9 mRNA into target cells, achieving efficient gene editing [47]. These advancements highlight the versatility and potential of LVs and the hence derived VLPs in gene therapy applications.

## 3. Pseudotyping: Acquiring the Right Coat

Achieving targeted gene delivery strongly depends on the choice of envelope glycoproteins displayed on LVs since they play a critical role in determining cell tropism and transduction efficiency. HIV-1 encodes its envelope glycoprotein, gp160, which is cleaved by cellular protease furin into gp120 and gp41. These proteins form trimers on the surface of HIV-1 particles, enabling the virus to infect CD4+ cells through the interactions of gp120 with the CD4 receptor and co-receptors, CCR5 or CXCR4 [48,49]. Importantly, it has been well-established that HIV-1 particles can also incorporate heterologous viral glycoproteins, such as the vesicular stomatitis virus (VSV) glycoprotein (G), which replaces the native gp120-gp41 through a process called pseudotyping [50]. This confers to the virus the ability to infect cells lacking CD4, significantly broadening its tropism. VSV-G has become the most widely used viral surface glycoprotein for pseudotyping LVs owing to its high fusogenic activity, exceptional stability, and broad tissue tropism, making it ideal for producing high-titer LVs suitable for clinical applications. For example, the first FDA-approved gene therapy drug, Tisagenlecleucel, uses VSV-G-pseudotyped LVs to express a CAR targeting CD19 [3].

VSV-G mediates viral entry by binding to the low-density lipoprotein receptor (LDLR), which is ubiquitously expressed on the surface of most cell types, conferring LVs with broad tropism [51,52]. This property has allowed VSV-G-pseudotyped LVs to successfully transduce a wide variety of cell types, including blood hematopoietic CD34+ cells, skin fibroblasts, epithelial cells, and mesenchymal stem cells [53,54,55,56,57,58]. While this broad tropism represents an advantage for ex vivo applications, where specific cell types can be isolated, modified using LVs, and reintroduced into patients, it poses a challenge for in vivo applications, where the selective targeting of specific cell types is critical to avoid off-target effects.

To address this limitation, envelope glycoproteins from various other viruses have also been explored for LV pseudotyping, some of which allow for the effective targeted transduction of the desired cell types. For example, the glycoprotein of the lymphocytic choriomeningitis virus (LCMV) can efficiently pseudotype LVs, achieving titers similar to those obtained with VSV-G while exhibiting low toxicity [59,60]. An important advantage of LCMV-G-pseudotyped LVs is their preferential infection of glioma cells over normal neurons, making them a promising drug candidate for targeting human glioblastoma [61]. Indeed, pre-clinical studies have further supported their effectiveness in eradicating glioblastoma in animal models [62,63]. Similarly, the hemagglutinin (H) and fusion (F) proteins of the measles virus (MV) have been used to pseudotype LVs, generating high-titer particles [64]. Compared to VSV-G, MV H and F exhibit the enhanced transduction of lymphocytes (T and B cells) and dendritic cells, making them particularly effective for targeting malignant plasma cells in multiple myeloma [65,66,67,68,69]. Another notable example is the use of hepatitis C virus (HCV) glycoproteins E1 and E2 to pseudotype LVs, which allow the efficient transduction of hepatocarcinoma cells [70]. In a pre-clinical study, HCV E1/E2-pseudotyped LVs equipped with Cas9/gRNA targeting the kinesin spindle protein (KSP) gene were administered to orthotopic Huh7 mice via intraperitoneal injection [71]. These LVs successfully delivered Cas9/gRNA to hepatocellular carcinoma (HCC) tumors in the liver, halting tumor growth, further emphasizing the potential of engineering LV tropism for cell-specific gene delivery in vivo [71]. In addition, envelope proteins from other viruses and engineered virus surface proteins have also been used to pseudotype LVs, which has been reviewed by others and will be further discussed in the following section [24,72,73,74].

## 4. Engineering Viral Tropism to Achieve Targeted Delivery

In addition to discovering heterologous viral glycoproteins that can efficiently pseudotype LVs and provide specific cell tropism, protein engineering has also been actively performed to create the desired tropism. In principle, the goal for the genetic modification of viral glycoproteins is to optimize the two key functions required for successful viral entry: high-affinity binding to cell surface receptors to enable specific LV attachment and the subsequent membrane fusion to deliver the genetic payload into the cytoplasm [75]. Receptor binding often triggers the downstream conformational changes required for membrane fusion. This fusion process depends on the controlled exposure and insertion of the viral fusion peptide or fusion loop into target membranes. The viral fusion peptide, with its highly hydrophobic amino acid sequence, remains hidden and protected until the viral glycoprotein engages its receptor. For some viruses (e.g., HIV-1 and paramyxoviruses), receptor binding alone triggers envelope glycoprotein conformational changes that expose the fusion peptide, though the specific mechanisms differ [49,76]. In contrast, other viruses rely on endocytosis into endosomes and lysosomes, where cues such as low pH (e.g., influenza virus, VSV, Sindbis virus) or cleavage by lysosomal proteases (e.g., Ebola virus) induce the necessary conformational changes for fusion [77,78,79]. Following insertion of the fusion peptide into the cellular membrane, the viral glycoprotein undergoes additional conformational changes to further shorten the distance between the two membranes, facilitating lipid mixing, membrane hemi-fusion, and complete fusion of the virus and host membranes.

From a protein engineering perspective, designing a membrane fusion machinery de novo that can precisely execute these sequential events presents a notable challenge. Instead, efforts to date have focused on designing targeting ligands to modify the tropisms of existing viral glycoproteins, particularly those that have been shown to efficiently pseudotype LVs. In essence, the targeting ligand must exhibit high specificity and affinity for a specific cell surface receptor, enabling the LVs to bind selectively to the target cells. Among the most commonly used targeting ligands are recombinant antibody molecules including single-chain variable fragments (scFvs), designed ankyrin repeat proteins (DARPins), and cytokines. scFvs, derived from the variable regions of antibody heavy and light chains connected by a short, flexible peptide linker, retain antigen-binding specificity and have been extensively used to retarget LVs pseudotyped with VSV-G or MV glycoproteins [80]. For example, scFvs targeting CD8 or CD19 have allowed for specific transduction of T and B cells, respectively, and are utilized in both research and therapeutic applications [81,82,83,84]. DARPins, genetically engineered antibody mimetics derived from cellular ankyrin proteins, exhibit high affinity and specificity, providing alternatives to scFvs due to their increased stability and resistance to aggregation [85,86,87,88]. They have been successfully used to redirect LV tropism to a variety of diverse cell types, such as HER2/neu positive cancer cells or T lymphocytes [87,89]. Cytokines have also been demonstrated to serve as LV targeting ligands, taking advantage of their natural binding affinity for their cognate receptors expressed on specific cell populations, such as immune cells or cancer cells [83,90]. These targeting ligands are typically displayed on the surface of LV particles as genetic fusions or via bridging proteins and allow for the enhanced precision of LV-mediated gene delivery, reducing off-target effects and improving the efficacy and safety of gene therapies for a wide range of applications. However, their binding alone does not complete cell entry and transduction, for which viral glycoproteins must be present.

**Figure 2:**
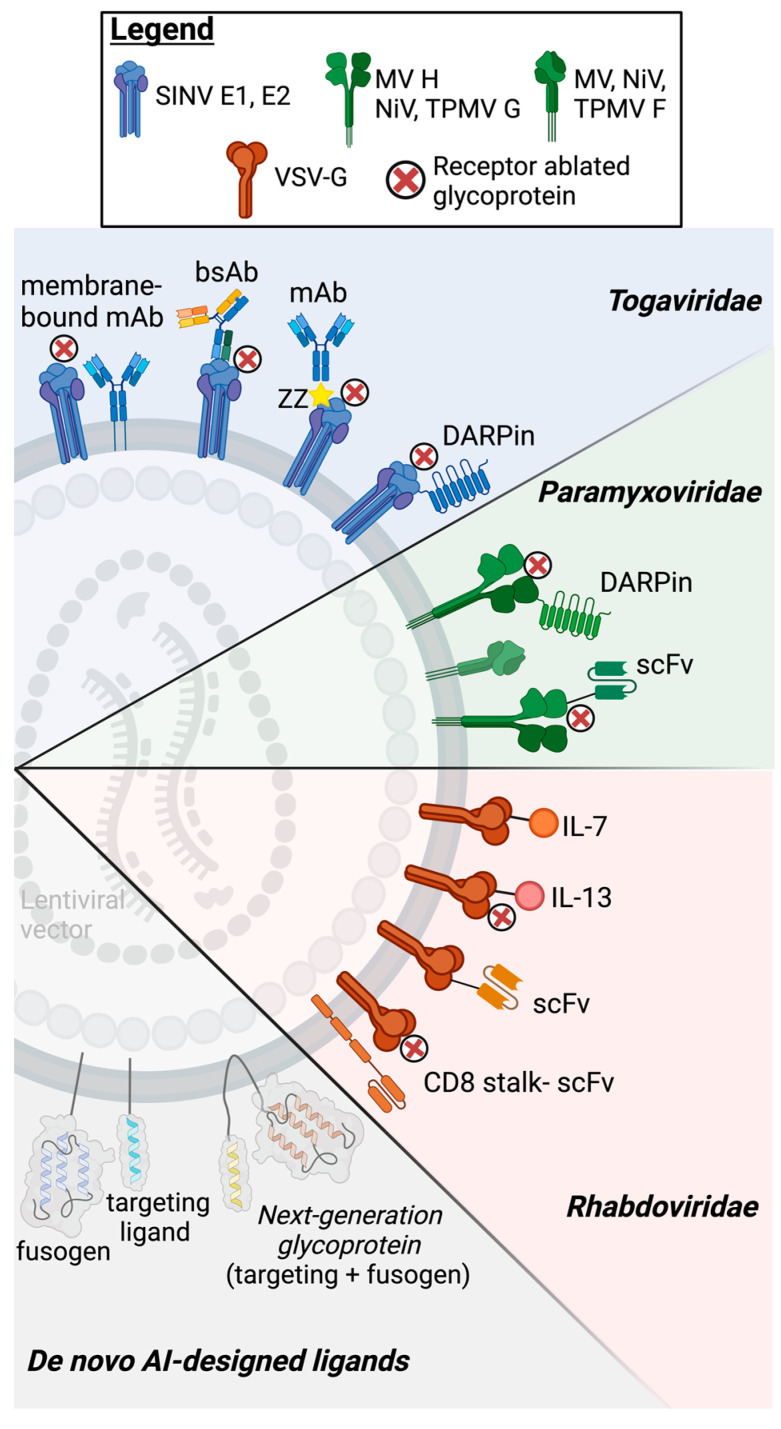
Targeting approaches for lentiviral vector pseudotyping. Schematic representation of LV pseudotyping strategies using heterologous glycoproteins, including those from Togaviridae (SINV), Paramyxoviridae (MV, NiV, and TPMV), and Rhabdoviridae (RSV). Various targeting ligand approaches are depicted, including the use of monoclonal antibodies (mAbs), bispecific antibodies (bsAbs), designed ankyrin repeat proteins (DARPins), single-chain variable fragments (scFvs), and cytokine-based ligands (IL-7, IL-13). Receptor-ablated glycoproteins are also employed to enhance specificity. Also depicted are de novo AI-designed ligands for improved LV targeting. Created in BioRender.com; accessed on 3 May 2025.

A number of heterologous viral glycoproteins have been demonstrated to efficiently pseudotype LVs, as discussed in earlier sections of this review [36,91]. Before attaching these ligands to viral glycoproteins, an engineering step is necessary: the ablation of natural receptor binding while preserving membrane fusion activity. This is because if their natural receptor recognition is not modified, cells expressing the natural entry receptor will be transduced along with target receptor-positive cells, even if they lack the target receptor. This poses a significant challenge for in vivo applications, as the systemic expression of natural receptors can lead to the off-target transduction of tissues and cells, thus compromising the precision and safety of therapeutic gene delivery. For example, it has been demonstrated that while LVs co-displaying unmodified VSV-G with scFv fragments enhance the transduction of target cells, off-target cells expressing the natural LDLR receptor remain transduced [72,92]. This engineering step has been successfully accomplished for viral envelopes where the function of attachment and fusion is separated on two glycoproteins, such as paramyxoviruses and togaviruses [93,94,95]. In contrast, destroying natural receptor binding while preserving membrane fusion has proven challenging for glycoproteins like retroviral envelope proteins where receptor binding and membrane fusion functions are combined in a single polypeptide and are intricately connected. A notable exception is VSV-G, whose conformational change is triggered solely by a low pH and is independent of receptor binding. This unique property enables the development of a bipartite fusion system: a separate targeting ligand mediates cell attachment, while a receptor-binding-deficient VSV-G mutant retains the ability to drive membrane fusion under a low pH in endo-lysosomes. The remainder of this section will explore the advancements in targeting strategies using these glycoproteins, organized by virus family, and highlight the engineering approaches to enhance the specificity and efficiency in gene delivery, focusing on the unique properties and challenges of each glycoprotein family (Figure 2 and Table 1).

### 4.1. Pseudotyping with Paramyxoviruses

Paramyxoviruses, namely the measles virus (MV), Nipah virus (NiV), and Tupaia virus (TPMV), have emerged as promising candidates for LV pseudotyping due to their unique glycoprotein structure which separates receptor-binding and fusion functions into distinct proteins. While MV uses the hemagglutinin (H) protein for attachment, NiV and TPMV utilize the glycoprotein (G). In all cases, the fusion protein (F) facilitates pH-independent membrane fusion at the plasma membrane [93]. This separation offers advantages for protein engineering, as modifying the attachment protein does not disrupt the fusion mechanism, allowing for targeted adaptations. For instance, it has enabled the development of targeted LV delivery by retargeting the receptor-binding protein and fusing it with targeting ligands such as scFvs or DARPins, while preserving the effective fusogenic activity [89,96].

Early work focused on MV glycoproteins, although their broad natural tropism—mediated by the native receptors CD46, signaling lymphocyte activation molecule (SLAM), and Nectin-4, which are expressed on a wide range of human cells—initially posed significant challenges for their use in LV pseudotyping [97,98]. The first successful demonstration of MV retargeting overcame this challenge by ablating the native receptor-binding domains of the MV H protein through key point mutations (Y481A, R533A, S548L, F549S) and fusing the resulting protein with scFvs specific for CD38 or EGFR [99]. This engineering allowed for selective viral entry exclusively through the targeted receptors in mice bearing CD38- or EGFR-positive human tumor xenografts, effectively eliminating the interactions with native receptors [99]. This approach was later adapted for LVs, using MV glycoproteins displaying scFvs against EGFR or CD20 [100]. Key point mutations in H and cytoplasmic tail truncations allowed the precise transduction of EGFR-expressing cells and CD20-positive lymphocytes [100]. Subsequent studies have expanded the targeting to diverse cell types, including neurons, endothelial cells, immune cells, and hematopoietic stem cells [81,101,102]. For instance, a CD8-specific scFv was developed to enable LV-mediated gene delivery to CD8+ T cells, both in vitro and in vivo, delivering melanoma-reactive T-cell receptors (TCRs) that effectively killed melanoma cells [81]. Similarly, CD90-targeted LVs were designed to specifically transduce HSCs, altogether demonstrating the versatility of MV-LVs for targeted gene delivery in cancer immunotherapy and regenerative medicine [102].

Further studies in retargeting strategies using MV glycoproteins have also leveraged DARPins. Notably, chimeric MV H-DARPins targeting HER2/neu—a receptor highly overexpressed in many cancers, such as breast and ovarian—redirected membrane fusion of pseudotyped LVs in xenograft mouse models, demonstrating their in vivo potential [87]. CD4- and CD8-specific DARPins also enabled LV retargeting in murine models, facilitating the delivery of HIV-1 viral entry inhibitor C46 peptide to CD4+ T cells or a CD19-specific CAR to CD8+ T cells [89,96]. These approaches effectively inhibited HIV-1 infection or eliminated B lymphocytes and lymphoma cells, with important implications for both HIV-1 and cancer therapy.

Despite these advancements, challenges remain with MV-LVs, including low scalable production and neutralization by pre-existing antibodies induced via widespread MV vaccination [103,104,105]. Alternative paramyxovirus glycoproteins from NiV and TPMV have therefore also been explored for LV pseudotyping. TPMV glycoproteins, originating from an animal paramyxovirus unrelated to any human pathogen, have been suggested to offer significant immune evasion from pre-existing antibodies [106]. These glycoproteins have been successfully engineered to display scFvs, demonstrated by CD20-targeted TPMV-LVs able to selectively transduce CD20-positive cells, including quiescent primary human B cells [106]. However, inefficiencies in vector production have limited the potential of this system for therapeutics. In contrast, NiV-based vectors have shown promise, benefiting from virtually no neutralization in human populations, along with up to 100-fold higher production titers [83]. NiV glycoproteins can additionally be engineered to target a variety of cell surface receptors, including EpCAM, CD20, and CD8, with high specificity, further underlining their potential for clinical settings, particularly where pre-existing immunity poses a barrier to MV-LVs [82].

### 4.2. Pseudotyping with Togaviruses

The Sindbis virus (SINV), the most commonly used togavirus for LV pseudotyping, features glycoproteins with distinct receptor-binding (E2) and fusion (E1) functions, similar to MV [94,95]. The SINV uses heparan sulfate proteoglycans (HSPGs) as attachment factors to concentrate the virus on the cell surface, while the 67 kDa laminin receptor (67LR) and NRAMP2 (DMT1) act as true receptors to mediate viral entry. The wide expression of 67LR and DMT1 confers broad tropism to the SINV, challenging cell-type-specific gene delivery via LV pseudotyping [94,95].

Important efforts to address this limitation involved engineering SINV particles to display the Fc-binding region of protein A (ZZ domain) from *Staphylococcus aureus* within E2 [107]. Early studies showed that this modification allowed for targeted delivery to human lymphoblastoid cells using monoclonal antibodies (mAbs) against cell surface antigens, achieving productive infection via EGFR [107]. This approach was adapted for LVs using SINV envelopes with ablated native receptor tropism, termed m168, and the ZZ domain, enabling the efficient transduction of CD4+ cells, and successful in vivo targeting of P-glycoprotein-expressing metastatic melanoma in murine models following intravenous injection [108,109,110]. Further refinements redirected SINV-based LVs via alternative adaptors, such as the biotin-adaptor peptide or integrin-targeting peptides, offering more stable conjugation of targeting molecules and broadening the application of SINV-LVs for experimental and clinical settings [111,112]. Similar strategies have been adapted by co-expressing the SINV E2 glycoprotein and targeting molecules, such as membrane-bound IgG antibodies recognizing CD20, on the LV surface without fusing them into a single polypeptide chain. This approach preserves the functional integrity of both components while enabling the flexible retargeting and efficient transduction of CD20+ B cells, both in vitro and in vivo [113,114,115].

Building on these efforts to refine SINV-LVs for targeted gene delivery, recent advancements have further enhanced their specificity and adaptability. For instance, a novel “plug-and-play” strategy incorporates a disulfide bond-forming protein (PDZ1) into a binding-deficient SINV E2 envelope, while its peptide ligand (TEFCA) is fused to cell-targeting proteins, such as DARPins, enabling the covalent conjugation of targeting molecules to SINV-LVs via a disulfide bond [116]. This approach has been shown to specifically redirect LVs to HER2+, although its in vivo performance remains to be validated [116]. The use of bispecific antibodies (bsAbs) binding both SINV E2 and receptors on target cells has been further suggested to improve SINV-LV specificity. Initially demonstrated to selectively transduce HER2-positive cells, it has also been extended to in vivo CAR-T cell engineering, where bsAb-redirected SINV-LVs could efficiently generate functional CAR-T cells in immunodeficient mice, offering a promising platform for personalized cancer immunotherapy [117,118]. Therefore, while the further optimization of targeting specificity and efficiency in complex in vivo environments is ongoing with SINV-LVs, significant progress has been made towards precise and flexible gene delivery for research and therapeutic applications.

### 4.3. Pseudotyping with Rhabdoviruses

Among the rhabdovirus glycoproteins used for LV pseudotyping, the VSV-G protein remains the most widely adopted due to its stability and high fusogenicity [50]. Other members like rabies virus glycoprotein (RV-G) have been reviewed elsewhere for specialized applications, particularly for their use in RV-G/VSV-G chimeric proteins (FuGs) enabling retrograde neuronal transport in rodent and non-human primate brains [119,120]. Cocal vesiculovirus G protein has also been shown to efficiently pseudotype LVs for hematopoietic cell transduction, offering the added advantage of resistance to inactivation by human serum [121]. The broad native tropism of VSV-G however, mediated by ubiquitous LDLR expression, has driven extensive efforts to achieve targeted gene delivery [24,122]. Initial attempts to alter the infection profile of VSV-G involved inserting scFvs into its N-terminal region. They first utilized an scFv specific for human Major Histocompatibility Complex class I (MHC-I), which enabled LVs pseudotyped with the engineered VSV-G to specifically target human cells expressing MHC-I [123]. While this approach demonstrated the feasibility of retargeting VSV-G through scFv fusion, the transduction efficiencies were much lower than those of wild-type VSV-G due to the reduced fusogenic activity. Subsequent studies optimized this strategy for in vitro use by combining scFv-VSV-G fusion activity with poloxamer-based adjuvants, achieving the efficient transduction of difficult-to-transduce cells like CD30+ lymphoma cells, CD34+ hematopoietic stem cells, EGFR+ tumor cells, and T cells [92,124].

Significant progress in VSV-G retargeting came with the knowledge that, unlike many viral glycoproteins where receptor binding triggers conformational changes, VSV-G relies solely on a low pH to drive membrane fusion. This unique property allows for the separation of receptor binding from fusion, making VSV-G highly adaptable for engineering targeted gene delivery systems like those in paramyxoviruses and togaviruses. However, this insight was only successfully implemented with the resolution of the structure of VSV-G with LDLR, which has finally allowed researchers to ablate VSV-G’s natural receptor binding while preserving its membrane fusion activity via precise mutagenesis [52]. Notably, a mutant VSV-G (VSVGmut) with K47Q and R354A mutations has been developed, which loses LDLR binding and natural tropism but retains fusion activity, making it ideal for cell-specific retargeting of LVs [83]. By co-expressing VSVGmut with cell-specific targeting molecules, such as anti-CD19 or cytokines like IL-13 that are presented with a transmembrane protein scaffold similar to the chimeric antigen receptor (CAR), pseudotyped LVs have been successfully redirected to specific cell populations [83,84]. For instance, LVs displaying both VSVGmut and an anti-CD19 scFv specifically transduced CD19-positive Raji B cells but not CD19-negative Jurkat T cells [84]. In support of this cell targeting role of anti-CD19 antibodies, LVs carrying anti-CD19 CARs were shown to unintentionally transduce malignant B cells [125]. A modular platform, termed DIRECTED (Delivery to Intended REcipient Cells Through Envelope Design), has further expanded the targeting capabilities using VSVGmut by incorporating multiple strategies to recruit or immobilize antibodies on the viral envelope, including a chimeric antibody-binding protein AG (pAG) or a SNAP-tag system, providing the potential for programmable, cell-specific gene delivery tailored to specific applications [126].

The use of VSVGmut holds promise for both research and clinical applications. In research settings, it has proven valuable for high-throughput interaction mapping studies, where VSVGmut-based LVs facilitated the precise identification of antigen–receptor interactions of B and T cells [83]. Clinically, the use of this approach has revealed important potential for cell-specific targeting of LVs encapsulating CRISPR–Cas9 and guide RNA [127]. By employing modified VSVGmut and scFv fragments targeting CD3 and CD4, this strategy enabled the precise delivery of the CAR gene into T cells and the expression of anti-CD19 chimeric antigen receptors (CARs) [127]. In humanized mouse models, these engineered CAR T cells effectively targeted and eliminated CD19+ B cells, even at a low level of T cell transduction (1.5%) [127]. Nevertheless, VSVGmut-based strategies continue to show great potential for both experimental and therapeutic applications, particularly in advancing next-generation in vivo CAR T cell therapies. For instance, the VSVGmut-based bipartite fusion system has been incorporated into the in vivo BCMA CAR-T drug candidate ESO-T01 for multiple myeloma, developed by EsoBiotec (recently acquired by AstraZeneca), which entered clinical trials at the end of 2024 [128]. 

**Table 1:** Targeting ligands for LV pseudotyping and their applications. Viral glycoproteins engineered alongside each targeting ligand, examples of targets and cell types, potential applications, and corresponding references are indicated. Asterix [*] symbol indicates ablation of native receptor binding in viral glycoprotein.

Targeting Ligand	Viral Glycoprotein	Examples	Target Cell Types	Potential Applications	References
**Antibodies** **(mAb, bsAb)**	SINV E2 *	membrane-bound anti-CD20 mAb	B lymphocytes	Cancer immunotherapy	Yang, L. et al. [113]; Ziegler, L. et al. [114]; Lei, Y. et al. [115]
	SINV E, E2 displaying the ZZ domain *	anti-CD4 mAb	CD4+ T cells	Immunomodulation	Morizono, K. et al. [108]; Liang, M. et al. [109]
		anti-P-glycoprotein	Metastatic melanoma cells	Cancer immunotherapy	Morizono, K. et al. [110]
	SINV E, E2 *	anti-E2xHER2 bsAb	Cancer cells (breast, ovarian, gastric)	Cancer immunotherapy	Parker, C.L. et al. [117]
		anti-E2xCD3 bsAb	T cells	Immunomodulation, CAR-T cell therapy	Huckaby, J.T. et al. [118]
**scFvs**	Measles (MV) H *	EGFR-specific scFv	Tumor cells	Cancer immunotherapy	Funke, S. et al. [100]
		CD20-specific scFv	B lymphocytes	Cancer immunotherapy	Funke, S. et al. [100]; Anliker, B. et al. [101]
		CD8-specific scFv	CD8+ T cells	CAR-T cell therapy	Zhou, Q. et al. [81]
		CD105-specific scFv	Endothelial cells	Vascular targeting	Anliker, B. et al. [101]
		GluA-specific scFv	Neurons	Neuromodulation	
		CD90-specific scFv	Hematopoietic stem cells (HSCs)	Regenerative medicine	Berckmueller, K. et al. [102]
	Tupaia (TPMV) G	CD20-specific scFv	B lymphocytes	Cancer (lymphoma) immunotherapy	Enkirch, T. et al. [106]
	Nipah (NiV) G *	EpCAM-specific scFv	Tumor cells	Cancer immunotherapy	Bender, R.R. et al. [82]
		CD8-specific scFv	CD8+ T cells	CAR-T cell therapy	
		CD20-specific scFv	B Lymphocytes	Cancer (lymphoma) immunotherapy	
	VSV-G	anti-MHC-I scFv	Nucleated cells	Immunomodulation	Dreja, H. et al. [123]
		anti-CD30 scFv	Lymphocytes	Cancer (lymphoma) immunotherapy	Anastasov, N. et al. [124]
		anti-CD34 scFv	Hematopoietic stem cells (HSCs)	Regenerative medicine	
		anti-EGFR scFv	Tumor cells	Cancer immunotherapy	Höfig, I. et al. [92]
	VSV-G * (VSVGmut)	anti-CD19 scFv	B lymphocytes	Cancer (lymphoma) immunotherapy	Yu, B. et al. [84]
		anti-CD3 and CD4 scFv	T cells	CRISPR-Cas9 delivery CAR-T cell therapy	Hamilton, J.R. et al. [127]
**DARPins**	Measles (MV) H *	HER2/neu-specific DARPin	Cancer cells (breast, ovarian, gastric)	Cancer immunotherapy	Münch, R.C. et al. [87]
		CD4-specific DARPin	CD4+ T cells	HIV entry inhibition	Zhou, Q. et al. [96]
		CD8-specific DARPin	CD8+ T cells	CAR-T cell therapy	Michels, A. et al. [89]
	SINV E2 *	HER2/neu-specific DARPin	Cancer cells (breast, ovarian, gastric)	Cancer immunotherapy	Kasaraneni, N. et al. [116]
**Cytokines**	VSV-G	membrane-bound IL-7	T cells	Immunotherapy CAR-T cell therapy	Verhoeyen, E. et al. [90]
	VSV-G * (VSVGmut)	membrane-bound IL-13	Immune cells	Cancer immunotherapy	Dobson, C.S. et al. [83]

## 5. Overcoming Immune Barriers to LV Transduction

Pre-existing immunity in the human population can be a barrier to the effectiveness of viral vector-based gene therapy. It has been well documented that the relatively high prevalence of adenovirus and AAV infections and hence the pre-existing anti-adenovirus capsid antibodies complicate the utility of adenovirus- and AAV-based vectors in gene therapy [23,129]. The same barrier can also occur to LVs, in the form of pre-existing antibodies as well as host intrinsic immunity [130].

### 5.1. Host Adaptive Immunity as a Barrier to LV Transduction

Since LVs are derived from HIV-1, pre-existing anti-HIV-1 adaptive immunity in people living with HIV (PLWH) may recognize and neutralize LV vectors. Therefore, the use of LV-based gene therapy for PLWH requires careful evaluation before being deployed clinically [131]. A second barrier is adaptive immunity targeting the heterologous viral envelope proteins used to pseudotype LV particles. For individuals who have been previously exposed to the virus whose envelope is used to pseudotype LVs, the pre-existing anti-viral envelope antibodies are expected to recognize and quickly clear these LVs, reducing their therapeutic efficacy. Similarly, population immunization against pathogenic viruses also limits the use of their envelope proteins to pseudotype LVs. A prominent example is the measles virus envelope protein, which enables high-titer LV production but poses a significant barrier in gene therapy due to pre-existing anti-measles antibodies from widespread vaccination [132,133]. Beyond the pre-existing adaptive immunity from prior viral infections and/or vaccinations, an equally limiting obstacle is the adaptive immunity elicited by the LV administration itself. This anti-LV immunity can significantly reduce the efficacy of subsequent administrations that are often necessary to achieve optimal therapeutic outcomes [134]. The immune response targets both the viral surface envelope proteins and internal capsid proteins, with envelope-directed neutralizing antibodies posing the greatest threat to subsequent LV deliveries [135]. Importantly, this challenge is not unique to LVs but extends to all viral vector-based therapies, and the deleterious effect of viral envelope-specific humoral immunity on the outcome of gene therapy has been reported in trials involving human subjects [136]. One potential strategy to circumvent this limitation is to pseudotype LVs with heterologous viral envelopes for subsequent deliveries of the same transgene. This approach has been demonstrated as effective in a mouse study, where LVs pseudotyped with glycoproteins (G) from Cocal virus (COCV), Maraba virus (MARAV), and Piry virus (PIRYV) successfully evaded pre-existing humoral immunity against the G protein of the vesicular stomatitis virus Indiana strain used to pseudotype LVs in the initial round of gene therapy [137]. Immune responses to viral vectors have been extensively discussed in other reviews [138].

### 5.2. Host Intrinsic Immunity as a Barrier to LV Transduction

**Figure 3:**
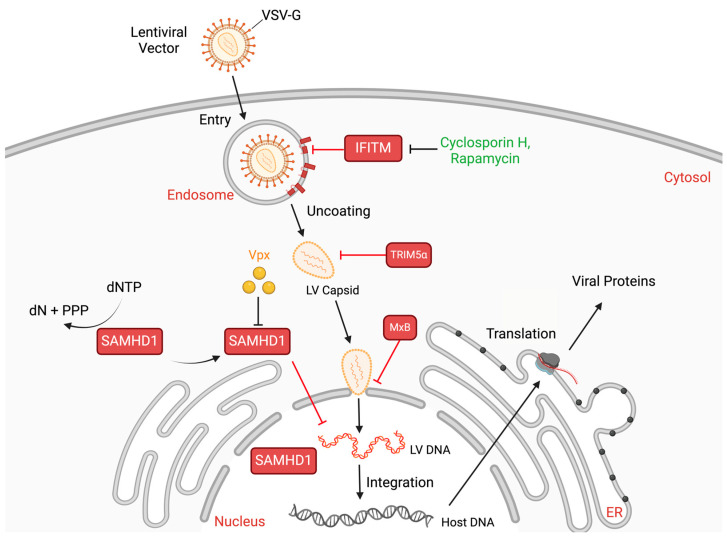
Host factors restricting lentiviral vectors from entry to DNA integration. Host restriction factors are presented in red boxes beside the viral replication steps that they target, including IFITM at entry, TRIM5a at viral capsid, MxB at nuclear entry of viral DNA, and SAMHD1 at viral DNA synthesis. Strategies to mitigate these host restriction factors are also indicated such as viral Vpx protein for SAMHD1 and drugs like CsH and Rapamycin for IFITMs. Created in BioRender.com; accessed on 3 May 2025.

In addition to host adaptive immunity, it is expected that host restriction mechanisms against HIV-1 would also hinder the transduction efficiency of LVs [139,140]. Indeed, a battery of host factors have been discovered to inhibit HIV-1 infection and viral production through diverse mechanisms [141]. The beginning of this impactful line of research was marked by the discovery that apolipoprotein B mRNA editing enzyme catalytic subunit 3G (APOBEC3G) restricts HIV-1 infection through deaminating cytidine to uridine in the minus-strand viral RNA during reverse transcription, resulting in hypermutations that render the viral genome non-infectious [142,143,144]. This landmark discovery was followed by the identification of additional host factors that inhibit HIV-1 infection by targeting distinct steps of viral replication. These mainly include Tripartite motif-containing protein 5 (TRIM5α), Tetherin, SAM and HD domain containing deoxynucleoside triphosphate triphosphohydrolase 1 (SAMHD1), Myxovirus resistance protein B (MxB), Interferon-induced transmembrane (IFITM) proteins, and Serine incorporator 5 (SERINC5) [145,146,147,148,149,150,151,152]. Some of these restriction factors operate in virus-producer cells, and can therefore be circumvented during LV production by using cell lines such as HEK293T, which lack the expression of these factors, including APOBEC3G, Tetherin, and SERINC5 [142,147,152,153,154,155,156]. However, LVs are inevitably subjected to restriction factors that target steps from HIV-1 entry through viral DNA integration (Figure 3). If these factors are expressed in the target cells, strategies must be implemented to help LVs evade their inhibition.

Monkey TRIM5α strongly inhibits HIV-1 infection by targeting the viral capsid core that is delivered into the cytoplasm of the target cells after successful virus entry, leading to the degradation of the viral core and genome complex [145,146]. Fortunately, HIV-1 has evolved to resist human TRIM5α by altering its capsid protein [157]. MxB also targets the HIV-1 capsid and inhibits the entry of the viral capsid and DNA into the nucleus. However, HIV-1 can evade MxB-mediated inhibition through mutating its capsid sequence [150,153,154]. Another major restriction factor, SAMHD1, is a dNTP triphosphohydrolase that inhibits viral replication in non-dividing cells by depleting the intracellular dNTP pool, thereby blocking the reverse transcription of LV transgene RNA [158]. One solution to counteract SAMHD1 restriction involves the HIV-2 accessory protein, Vpx, which can be incorporated into HIV-1 particles through its interaction with viral Gag protein. Following virus entry, Vpx can bind to SAMHD1, causing SAMHD1 ubiquitination and degradation through recruiting the ubiquitin ligase substrate receptor, DCAF1 [155,156]. The degradation of SAMHD1 elevates the levels of the dNTP pool and therefore restores viral reverse transcription.

Rescuing LVs from inhibition by IFITM proteins can be challenging when different viral glycoproteins are used for pseudotyping, primarily due to the broad spectrum of viruses affected by IFITM-mediated restriction [151,159,160]. IFITM proteins are a family of small, ubiquitously expressed proteins that play a pivotal role in the host’s antiviral defense by inhibiting the entry of numerous viruses [151,159,160]. The primary members—IFITM1, IFITM2, and IFITM3—are upregulated in response to interferon signaling and are predominantly localized in the endo-lysosomal membranes and the plasma membrane [161,162]. Their localization enables them to effectively block the fusion of viral membranes with host cellular membranes, thereby preventing the release of viral genomes into the cytoplasm and blocking the subsequent replication processes. Notably, IFITM proteins have been shown to alter the biophysical properties of cellular membranes, increasing the membrane rigidity and curvature, which hinders membrane lipid mixing. This alteration effectively traps the virus in endo-lysosomal compartments and targets it for degradation [163,164,165,166]. Nevertheless, while the broad antiviral activity of IFITM proteins provides significant benefits to the host, it presents consequential challenges when LVs are used for gene therapy. For example, it has been reported that IFITM proteins strongly inhibit the entry of LV vectors pseudotyped with VSV-G [167,168].

One strategy that has been explored to overcome IFITM inhibition is to lower the levels of IFITM proteins in the target cells. Although IFITM expression is often stimulated by interferons, certain cell types constitutively express high levels of IFITM, including stem cells which are often the target cells of gene therapy [169]. To maintain their pluripotent state, stem cells do not respond to cytokines and interferons and instead protect themselves from viral infections by constitutively expressing a subset of antiviral interferon-stimulated genes (ISGs), including IFITM proteins. Studies have shown that when these constitutively expressed IFITM proteins are knocked down, stem cells become highly susceptible to infection by viruses such as the Zika virus (ZIKV), West Nile virus (WNV), Yellow Fever virus (YFV), Dengue virus (DENV), Respiratory Syncytial virus (RSV), Influenza A virus (IAV), and Newcastle Disease virus (NDV) [170]. Not surprisingly, the high expression of IFITM3 in hematopoietic stem cells (HSCs) limits the efficiency of LV transduction [171]. One promising approach for diminishing ISG levels involves the use of epigenetic regulators to transiently suppress ISG expression. For example, the histone deacetylase inhibitor, Quisinostat, has been shown to significantly enhance the LV transduction in HSCs without compromising their ability to engraft and differentiate. By altering the chromatin landscape, Quisinostat reduces the expression of antiviral ISGs, thereby creating a more permissive cellular environment for LV vector integration [172]. Alternative strategies to enhance the LV transduction efficiency involve the transient modulation of key signaling pathways, such as the mitogen-activated protein kinase/extracellular signal-regulated kinase (MAPK/ERK) pathway. For instance, inhibiting the MAPK/ERK pathway has been shown to improve the conversion of T cells into chimeric antigen receptor (CAR) T cells [173]. Specifically, strategies have been explored to reduce the IFITM levels in stem cells. For instance, treatment with the resveratrol trimer has been reported to enhance the LV transduction efficiency in HSCs by downregulating the IFITM expression, thereby reducing the intrinsic antiviral state of these cells [174]. In addition, cyclosporine H (CsH) was reported to increase the LV transduction of HSCs by 10-fold through downregulating IFITM3 levels, which led to an increase in the gene editing efficiency in SCID-repopulating HSCs [175]. Additionally, the well-known immunosuppressant and mTOR inhibitor, Rapamycin, has been observed to cause IFITM3 degradation by endo-lysosomes, thus significantly enhancing the LV transduction of HSCs [171]. Both CsH and Rapamycin are deemed effective in promoting the ex vivo genetic modification of target cells with LVs, while their in vivo utility remains unclear.

In addition to lowering the levels of IFITM proteins in target cells, an alternative strategy is the use of heterologous viral glycoproteins that resist IFITM, as it is known that not all viral glycoproteins are susceptible to IFITM inhibition. For example, the Lassa virus and human parainfluenza virus type 3 (PIV-3) are completely resistant to IFITM inhibition [176,177]. More notably, studies have shown that IFITM2 and IFITM3 stimulate the entry of human coronavirus OC43 (HCoV-OC43), a virus causing the common cold [178]. While the mechanisms underlying this resistance and enhancement are not completely understood, one study showed that Lassa virus glycoprotein-mediated entry occurs in cellular membrane compartments that are devoid of IFITM3, thus avoiding this cellular inhibition [176]. While these IFITM-resistant viral glycoproteins may not be directly suitable or compatible for LV pseudotyping, a detailed insight into the molecular characteristics associated with their resistance may inform the rational design and selection of IFITM-resistant fusogenic proteins. Alternatively, directional evolution can be performed to select for IFITM-resistant VSV-G or other heterologous viral glycoproteins that have been successful in pseudotyping LVs. The feasibility of this approach is supported by the observation that the Env glycoproteins of some HIV-1 strains are sensitive to IFITM inhibition, while others are resistant, indicating that the same viral glycoprotein can change its susceptibility to IFITM inhibition by altering its amino acid sequence [179,180,181,182]. In support of this scenario, a proline-to-histidine substitution at position 681 (P681H) in the Spike glycoprotein of the SARS-CoV-2 Alpha variant (B.1.1.7) confers resistance to IFITM2 inhibition [183]. This mutation is located at the furin cleavage site, resulting in the enhanced cleavage efficiency of the spike protein, thereby facilitating more effective viral entry.

Beyond natural viral mutations, the existing knowledge about viral resistance to IFITM-mediated restriction presents a valuable opportunity to engineer more efficient LV envelopes. By identifying the structural and functional adaptations that allow certain viruses to evade or even exploit IFITM proteins, LV envelopes that are inherently resistant to IFITM inhibition can be engineered, thereby enhancing the transduction efficiency of the target cells with high IFITM levels. This approach is particularly relevant for in vivo gene delivery, as it focuses solely on modifying the vector rather than systemic conditioning of the host, thus minimizing potentially severe side effects.

## 6. Emerging Directions for LV Targeting

The current LV pseudotyping strategy has relied on natural targeting ligands, such as antibodies or cytokines, to bind specific cell surface markers. This approach may rapidly change due to the unprecedented breakthroughs in artificial intelligence (AI)-assisted protein design. Pioneering work by Dr. David Baker’s group has established computational platforms for the de novo generation of synthetic ligands against a desired cell surface marker, overcoming the constraints of natural targeting ligands. For instance, AI-driven computational tools like RoseTTAFold diffusion (RFdiffusion) allow the design of proteins with precise, user-defined protein–protein binding properties [184]. The power of these tools has been demonstrated in diverse applications, including designing proteins that neutralize snake venom toxins and generating potent TNF receptor agonists/antagonists [185,186,187]. It is plausible that similar strategies could be applied to design LV ligands, offering unmatched specificity and modularity for gene delivery. For example, instead of scFvs, an AI-designed ligand can be attached to a transmembrane scaffold, thus guiding LVs to the target cells (Figure 2).

Beyond targeting, de novo protein design also has the potential to create novel, artificial fusogenic proteins. While the complex conformational changes required for membrane fusion present challenges, the recent successes in designing functional hydrolase enzymes demonstrate that highly specific biological functions can be engineered de novo [186]. A next-generation artificial fusogen could therefore combine targeting and fusion into a single molecule (Figure 2), similar to the modular approach applied with VSVGmut-scFvs, but with the added benefit of being fully customizable to meet the unique requirements of specific research and clinical applications.

Importantly, these synthetic proteins would offer multiple advantages over natural glycoproteins. Firstly, they could avoid the pre-existing antibody responses that often limit the therapeutic efficiency. Second, they could not only resist IFITM-mediated restriction but potentially harness host immune factors to enhance their entry, similar to how human coronavirus OC43 utilizes IFITM proteins to facilitate its infection. By integrating insights from naturally evolved viral adaptations and computational protein design, future LVs could achieve significantly improved transduction efficiency, expanding the potential of gene therapies for in vivo applications.

## 7. Conclusions

Both as powerful research tools and life-saving gene therapy medicines, LVs have undergone major advancements over the past decades, particularly in pseudotyping—from the use of heterologous viral glycoproteins to expand cell tropism to the design of ligands for targeted delivery. It is expected that AI-assisted protein engineering will further enable the possibility of the de novo design of synthetic ligands that precisely direct LVs to cells expressing specific marker proteins, significantly advancing the current modular design strategy that relies on antibodies or antibody mimetics like DARPins. The recent success of de novo enzyme design also raises the possibility of engineering novel viral fusion proteins that not only mimic the conformational changes and membrane fusion functions of native viral glycoproteins, but also enhance and introduce new capabilities, such as resisting host restriction by IFITM proteins. Given the versatility of LVs in delivering a range of diverse cargos, from transgenes to mRNAs to proteins, targeted delivery will be key to reaching their full potential, particularly in the development of in vivo gene therapy drugs.

## Data Availability

Not applicable.
